# GNPAT/USP30 Stabilizes DRP1 Protein to Promote Mitochondrial Fission and Functional Damage in COPD Progression

**DOI:** 10.1002/kjm2.70080

**Published:** 2025-07-25

**Authors:** Xin‐Gui Cheng, Yun‐Chan Liu, Fei Chen, Ji‐Wei Li, Xiao‐Zhou Yao, Qing‐Yun Chen

**Affiliations:** ^1^ Pulmonary and Critical Care Medicine Hainan Affiliated Hospital of Hainan Medical University Haikou China; ^2^ Pulmonary and Critical Care Medicine Hainan General Hospital (Hainan Affiliated Hospital of Hainan Medical University) Haikou China; ^3^ Department of Laboratory Affiliated to Shanghai Jiao Tong University School of Medicine Shanghai Children's Medical Center, Hainan Branch Sanya China

**Keywords:** chronic obstructive pulmonary disease, DRP1, GNPAT, mitochondrial fission, USP30

## Abstract

Our previous study revealed the role of glycerol phosphate O‐acyltransferase (GNPAT) in regulating chronic obstructive pulmonary disease (COPD). However, its further mechanisms remained unclear. In this study, COPD models were established by exposing mice to cigarette smoke particulates. H&E staining and immunohistochemistry assays were performed on COPD tissue. A549 cells were stimulated with 5% cigarette smoke extract (CSE) and transfected with GNPAT, ubiquitin‐specific protease 30 (USP30), and dynamin‐related protein 1 (DRP1) plasmids. Cell viability, cell apoptosis, lactate dehydrogenase (LDH) release, ATP production, and reactive oxygen species (ROS) levels were determined using commercial kits. Quantitative real‐time PCR and western blotting were used to evaluate mRNA and protein expression. Mitochondrial morphology was examined by transmission electron microscopy. A co‐immunoprecipitation assay determined the binding relationships among GNPAT, USP30, and DRP1. Our results showed that GNPAT and DRP1 were highly expressed in the COPD model mice. CSE promoted mitochondrial fission, mitochondrial dysfunction, and cell apoptosis, which were further enhanced by treatment with a mitochondrial fission inducer (TA9). GNPAT promoted mitochondrial fission, mitochondrial dysfunction, and cell apoptosis by enhancing DPR1 protein stability, which depended on USP30. DRP1 enhanced mitochondrial fission, mitochondrial dysfunction, and cell apoptosis, which were both reversed by GNPAT/USP30 inhibition. Collectively, our present study found that GNPAT recruited USP30 and stabilized DRP1, thereby mediating mitochondrial fission and mitochondrial dysfunction that contributed to cell apoptosis in COPD. This study suggests a promising therapeutic biomarker for COPD.

## 1 Introduction

Chronic obstructive pulmonary disease (COPD) is a common, treatable, and preventable heterogeneous respiratory disease featuring pulmonary airflow limitation and persistent respiratory symptoms [1, 2]. It is caused by airway and alveoli abnormalities, and has high rates of morbidity and disability [1]. COPD has a global prevalence of approximately 12% and is the 3rd leading cause of mortality worldwide [3]. With increasing industrialization and an aging population, the airway aggravation caused by various air pollutants continues to increase, along with the medical burden caused by slow‐blocking COPD [4]. Therefore, potential targets and novel therapies that rely on molecular mechanisms for COPD are greatly needed.

Cigarette smoke (CS) is one of the main risk factors for COPD, and approximately 80% of COPD patients develop the disease due to smoking [5]. Long‐term exposure to CS can cause persistent inflammation and immune responses in the airways and lungs that induce alveolar epithelial cell death, which is an essential process in the pathogenesis of smoking‐related COPD [6]. The protein encoded by glycerol phosphate O‐acyltransferase (GNPAT) is a crucial enzyme located in the peroxisome and involved in synthesizing ether phospholipids [7]. One of our previous studies demonstrated that cigarette smoke extract (CSE) inhibits SIRT4 expression, and reduced SIRT4 levels increase GNPAT acetylation and protein levels, subsequently promoting ferroptosis in alveolar epithelial cells [8]. However, the downstream mechanism of GNPAT in COPD has not been explored. Mitochondrial dynamics play an important role in regulating ferroptosis [9], and previous studies have confirmed the regulation of mitochondrial dynamics by GNPAT [10, 11]. Therefore, we explored whether GNPAT participates in COPD progression by regulating mitochondrial dynamics.

Mitochondria are essential organelles in cells that participate in various cellular processes such as oxidative phosphorylation, apoptosis, and calcium regulation [12]. Mitochondrial dynamics, that is, the fusion and fission processes of mitochondria, control the size, number, and distribution of mitochondria and further affect mitochondrial function and cell state. When mitochondria are damaged, cells upregulate the optic Atrophy 1 (OPA1) protein to initiate mitochondrial fusion, thereby compensating for metabolic abnormalities and maintaining mitochondrial function [13]. However, in the lung tissue of COPD patients, the number of mitochondria is increased, while the size and activity of mitochondria are decreased [4]. At the in vitro level, when cells are exposed to PM2.5 or CSE, mitochondrial fusion is blocked, mitochondrial fission increases, mitochondrial membrane potential is lost, and the levels of reactive oxygen species (ROS) increase [14, 15]. Those findings suggest a relationship between mitochondrial dynamics and COPD.

Dynamin‐related protein 1 (DRP1) is a GTPase that participates in mitochondrial fission and apoptosis [16]. Studies have found that CSE exposure is accompanied by increased DRP1 protein levels [17, 18]. However, surprisingly, when compared to non‐smokers, smokers have increased p‐DRP1 protein levels in their lung tissues but no significant changes in mRNA levels, suggesting that smoking may cause post‐translational regulation of the DRP1 protein [19]. It has been reported that GNPAT can recruit the deubiquitinase ubiquitin‐specific protease 30 (USP30) to stabilize the DRP1 protein in cancer [20]. Furthermore, previous research also revealed that DRP1 and mitochondrial fragmentation participate in ferroptosis [21, 22]. It is important to determine whether GNPAT recruits USP30 to stabilize the DRP1 protein, and thereby regulate mitochondrial fission and COPD progression. In this study, we investigated the roles of GNPAT and DRP1 in regulating COPD progression and explored the relationships among GNPAT, DRP1, and USP30. Our present study sought to identify a potential target or novel therapy for COPD.

## 2 Materials and Methods

### 2.1 Cell Culture and Intervention

A549 cells were purchased from the Institute of Cell Biology (Shanghai, China) and cultured in DMEM supplemented with penicillin/streptomycin and 10% FBS. All the cells were maintained at 37°C in a humidified tissue culture incubator with a 5% CO_2_ atmosphere.

Next, the A549 cells were treated with 5% CSE to mimic the COPD environment, and with 1 μM Tyrphostin A9 (a mitochondrial fission inducer) to explore the role of mitochondrial dynamics. Moreover, when the A549 cells reached 80%–90% confluence, they were transfected with overexpression plasmids (GNPAT, DRP1), an empty vector, shRNA targeting plasmids (sh‐GNPAT, shUSP30), and shNC by use of Lipofectamine 3000 (Thermo Fisher Scientific Inc. Waltham, MA, USA).

### 2.2 COPD Model Construction

A COPD mouse model was constructed in our previous study by using a smoking chamber [8], and the tissues from that study were used in this study. In brief, female C57BL/6 mice were fed a normal diet during a 1‐week adaptation period and then housed in the smoking chamber, during which time they were exposed to cigarette smoke for 2 h once a day for 6 months. Finally, the animals were anesthetized, and the lung tissues were isolated, collected, and stored at −80°C.

### 2.3 CSE Extract Preparation

Smoke from a non‐filtered cigarette was continuously drawn into 10 mL of sterile PBS by a negative pressure suction device to prepare a CSE solution. The CSE solution was then filtered using a 0.22 μm filter to remove cigarette residues and bacteria, and the resulting liquid was defined as a 100% CSE solution.

### 2.4 Hematoxylin–Eosin (H&E) and Immunohistochemistry (IHC) Staining

COPD tissues were collected and cut into 5 μm sections. H&E staining was performed using an H&E kit (Solarbio, China), according to the manufacturer's instructions.

For IHC analysis, the tissues were deparaffinized and rehydrated before being immersed in sodium citrate buffer for antigen retrieval. Next, the sections were incubated with primary antibodies against GNPAT and DRP1 at 4°C for 12 h. The tissue samples were then exposed to an HRP‐labeled goat anti‐rabbit IgG secondary antibody at 25°C for 40 min; after which, the positive area was observed with a microscope imaging system (Nikon, Tokyo, Japan).

### 2.5 Cell Counting Kit‐8 (CCK‐8) Assay

A549 cell activity was measured using the CCK‐8 assay (Solarbio). In brief, A549 cells were seeded, cultured at 37°C, and treated with CCK‐8 solution for 0, 6, 12, 24, and 36 h, respectively, and then incubated for another 2 h. Finally, the cell numbers were calculated by absorbance spectrometry at 450 nm.

### 2.6 Cell Apoptosis Analysis

An Annexin V‐FITC/PI detection kit (CA1020, Solarbio) was used for cell apoptosis analysis. The cells were collected and washed with ice‐cold PBS. Next, approximately 2 × 10^5^ cells were suspended and stained with Annexin V FITC and PI solutions for 20 min at 25°C in the dark. Flow cytometry (Beckman Coulter Inc. Brea, CA, USA) was used to analyze the stained cells for apoptosis induction.

### 2.7 Real‐Time Quantitative PCR (qPCR)

The levels of GNPAT, DRP1, and USP30 expression were evaluated in transfected cells. The primers for GNPAT, DRP1, and USP30 were designed and synthesized by Sangon (Shanghai, China), and were shown in Table 1. QPCR was performed by using a HiScript II One Step qRT‐PCR SYBR Green Kit (cat: #Q221‐01; Vazyme Biotech Co. Ltd. Nanjing, China) on an ABI 7900 qPCR system (Applied Biosystems, Foster City, CA, USA). Relative levels of gene expression were calculated using the 2^−ΔΔCt^ method, with *β*‐actin serving as a reference standard.

**TABLE 1:** Primer pairs used for quantitative RT‐PCR analysis.

Gene ID	Sequence (5′‐3′)
M‐Actin‐F	CAGCCTTCCTTCTTGGGTATG
M‐Actin‐R	GGCATAGAGGTCTTTACGGATG
M‐GNPAT‐F	AGCAGTGTGCTCCTCTATGC
M‐GNPAT‐R	ACTTGATGTCCCCTGGCTTG
M‐DRP1‐F	GAAAGGGCGGAGGAGAAGAG
M‐DRP1‐R	CCACTACGACGATCTGAGGC
H‐Actin‐F	CACTCTTCCAGCCTTCCTTC
H‐Actin‐R	GTACAGGTCTTTGCGGATGT
H‐GNPAT‐F	GCTCCTCTACTCGCTTTCCAA
H‐GNPAT‐R	GGCACAAAACCGAATGGCTC
H‐DRP1‐F	TCACCCGGAGACCTCTCATT
H‐DRP1‐R	TCTGCTTCCACCCCATTTTCT
H‐USP30‐F	ACTAGGGTCCATCCTCTGGG
H‐USP30‐R	GCACAAGCCCTTTTCTACGC

Abbreviations: F, forward primer; R, reverse primer.

### 2.8 Western Blot (WB) Analysis

The levels of GNPAT, DRP1, p‐DRP1, and USP30 protein expression were evaluated by western blotting. Standard sodium dodecyl sulfate‐polyacrylamide gel electrophoresis (SDS‐PAGE) was performed at 120 V. The PVDF membranes were washed and blocked with 5% powdered skimmed milk at 4°C overnight; after which, they were incubated for 1 h with primary antibodies against GNPAT (ab75060, Abcam, Cambridge, UK), DRP1 (ab184247, Abcam), phosphorylation (p)‐DRP1 (ab314755, Abcam), USP30 (ab314749, Abcam), and *β*‐actin (ab8227, Abcam). Next, the membranes were incubated with a horseradish peroxidase (HRP) secondary antibody‐labeled goat anti‐rabbit IgG. The PVDF membrane was then incubated with electrogenerated chemiluminescence (ECL) solution (ECL808‐25, Biomiga, San Diego, CA, USA) for 1 min and exposed to X‐ray film. *β*‐actin served as an internal reference band.

### 2.9 ATP Production Assay

ATP levels were measured using an ATP Assay Kit (Ab83355, Abcam, Cambridgeshire, UK) according to the manufacturer's instructions. In brief, A549 cells were centrifuged, and the supernatants were collected. The cell lysates and ATP reaction mixture (v:v = 1:1) were mixed and incubated for 30 min, and the absorbance at 570 nm was measured with a microplate reader (Infinite 200 Pro, TECAN).

### 2.10 Lactate Dehydrogenase (LDH) Activity Assay

LDH levels in supernatants were determined by using an LDH Cytotoxicity Assay kit (C0016, Beyotime Institute of Biotechnology) according to the manufacturer's instructions.

### 2.11 ROS Measurements

Total ROS was measured using a 2′, 7′‐dichlorofluorescein diacetate (DCFH‐DA) kit (Beyotime, Shanghai, China). Approximately 5 × 10^5^ cells were seeded into each well of six‐well plates and treated with 10 μmol/L DCFH‐DA diluted with serum‐free medium for 20 min at 37°C. The cells were then washed with a serum‐free medium, and the ROS levels in cells were analyzed by flow cytometry.

### 2.12 Transmission Electron Microscopy (TEM)

TEM was utilized to examine mitochondrial morphology. Cells were initially fixed with 4% glutaraldehyde and 1% citric acid. Subsequently, they were dehydrated in ethanol, embedded in Eponate 12 epoxy resin, and cut into semi‐thin sections using a LEICA EM UC7 Ultra‐Thin Slicer. The slides were double stained with uranyl acetate and lead citrate before examination by TEM (FEI Tecnai Spirit).

### 2.13 Co‐Immunoprecipitation (Co‐IP) Assay

The binding among GNPAT, DRP1, and USP30 was confirmed by a co‐IP assay that was performed using Absin Co‐IP kit (Absin, ab955, China). The transfected cells were washed with PBS and then lysed for 15 min. Afterward, they were incubated with specific antibodies or IgG at 4°C for 12 h. The following day, Protein A and G were added and incubated with rotation. The mixture was then centrifuged at 12,000 g for 1 min. Finally, the pellet was used for protein detection by western blotting (WB).

### 2.14 Statistical Analysis

Statistical analysis and graph generation were performed using GraphPad Prism 9 Software (San Diego, CA, USA). Depending on the experimental groups, either the unpaired Student's *t*‐test or one‐way ANOVA was used for statistical analysis. Data are presented as a mean value ± standard deviation (SD). A *p* < 0.05 was considered statistically significant.

## 3 Results

### 3.1 GNPAT and DRP1 Expression in the Lung Tissues of Normal and COPD Model Mice

**FIGURE 1:**
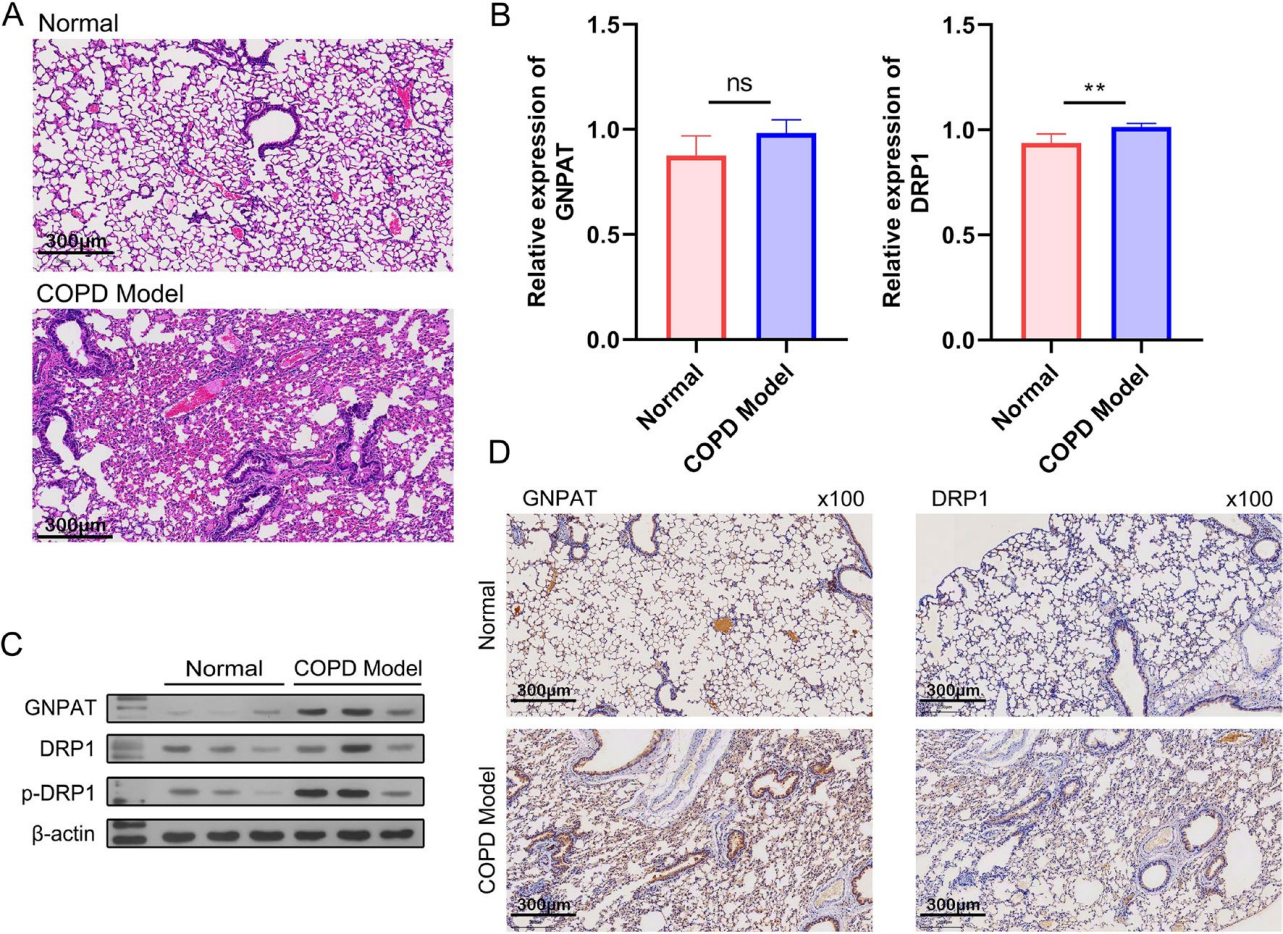
GNPAT and DRP1 expression in the lung tissues of normal and COPD model mice. A COPD mouse model was created by exposing mice to cigarette smoke. (A) H&E staining showed the tissue morphology in samples of Normal and COPD tissue. (B) A qPCR analysis evaluated the levels of GNPAT and DRP1 mRNA expression. (C) A WB analysis determined the expression levels of GNPAT, DRP1, and p‐DRP1 proteins, using *β*‐Actin as a reference standard. (D) IHC was used to evaluate the levels of GNPAT and DRP1 protein expression (×100). ns, not significant. **p < 0.01.

The C57BL/6 mice were assigned to two separate groups, and the COPD model was established. After model construction, the tissue morphology in the Normal and COPD groups was observed by H&E staining. Well‐defined alveolar structures with a uniform arrangement were observed in the Normal group, while thickened alveolar walls, partial tracheal obstruction, and reduced alveolar septa size were observed in the COPD model group (Figure 1A). Subsequently, the lung tissues were used for further studies. The levels of GNPAT and DRP1 mRNA were examined in the Normal and COPD groups, and no significant difference (GNPAT) or slight change (DRP1) were observed (Figure 1B). However, a WB analysis showed that GNPAT, DRP1, and p‐DRP1 were more highly expressed in the COPD group than in the Normal group (Figure 1C). IHC staining also showed higher expression levels of GNPAT and DRP1 in the COPD model group than in the Normal group (Figure 1D). These results suggested that GNPAT and DRP1 were highly expressed at the protein level but not at the corresponding mRNA level in COPD.

### 3.2 CSE Promoted Mitochondrial Fission and Functional Damage, Which Were Enhanced by the Mitochondrial Fission Inducer TA9

**FIGURE 2:**
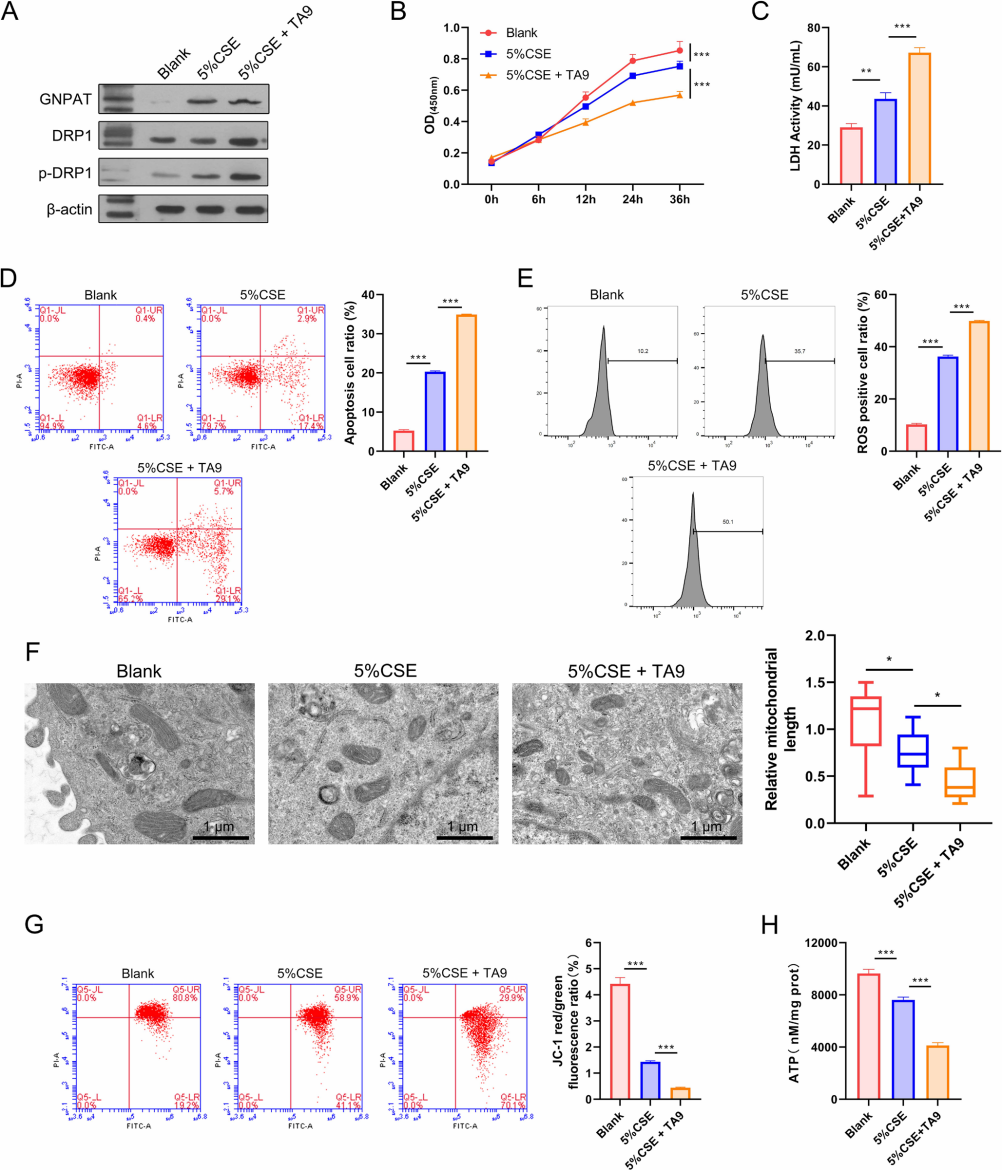
CSE promoted mitochondrial fission and functional damage, which were enhanced by the mitochondrial fission inducer, TA9. To verify the role of mitochondrial fission in CSE‐induced cell injury, A549 cells were treated with 5% CSE to mimic a COPD environment and then co‐incubated with 1 μM Tyrphostin A9 (a mitochondrial fission inducer). (A) A WB analysis determined the levels of GNPAT, DRP1, and p‐DRP1 protein expression, using β‐Actin as a reference standard. (B) The cellular activity of A549 cells undergoing treatment with 5% CSE and 5% CSE + TA9 at 0, 6, 12, 24, and 36 h was measured by the CCK‐8 assay. (C) LDH release was detected by the LDH activity assay. (D) Cell apoptosis was determined by flow cytometry. (E) The levels of total ROS were determined using a 2′, 7′‐dichlorofluorescein diacetate (DCFH‐DA) kit. (F) Mitochondrial morphology was examined by transmission electron microscopy. (G) Flow cytometry was used to assess mitochondrial membrane potential using JC‐1 dye. (H) ATP levels were measured using an ATP assay kit. **p < 0.01, ***p < 0.001.

To validate the role of mitochondrial fission in CSE‐induced cell injury, A549 cells were treated with 5% CSE to mimic the COPD environment and co‐incubated with 1 μM Tyrphostin A9 (TA9, a mitochondrial fission inducer). The levels of DPR1 and p‐DPR1 protein expression were measured in the Blank, 5% CSE, and 5% CSE + TA9 groups. Our data showed that GNPAT and p‐DRP1 protein expression were dramatically increased in the 5% CSE group, and p‐DRP1 expression was even further increased when TA9 was added (Figure 2A). Testing showed that both 5% CSE and TA9 significantly inhibited cellular activity when compared with the Blank group, with the 5% CSE + TA9 group showing enhanced inhibition when compared with the 5% CSE group at 36 h after treatment (Figure 2B). LDH release reached its highest level in the 5% CSE + TA9 group, followed by the 5% CSE group (Figure 2C), suggesting that CSE and TA9 promoted cellular damage. Flow cytometry revealed that treatment with 5% CSE and TA9 induced notable cell apoptosis and ROS production (Figure 2D,E). A TEM analysis showed that mitochondrial length was significantly reduced by 5% CSE and TA9 (Figure 2F). When compared with the Blank group, the JC‐1 red/green fluorescence ratio was significantly reduced by 5% CSE and then further decreased by 5% CSE + TA9 (Figure 2G), revealing reduced mitochondrial membrane potential caused by 5% CSE and TA9. Additionally, 5% CSE and TA9 significantly reduced the ATP levels when compared with the Blank group (Figure 2H). Taken together, these findings showed that CSE promoted mitochondrial fission and functional damage, and those effects were further enhanced by TA9.

### 3.3 GNPAT Increased DRP1 Protein Expression, and Promoted Mitochondrial Fission and Functional Damage

**FIGURE 3:**
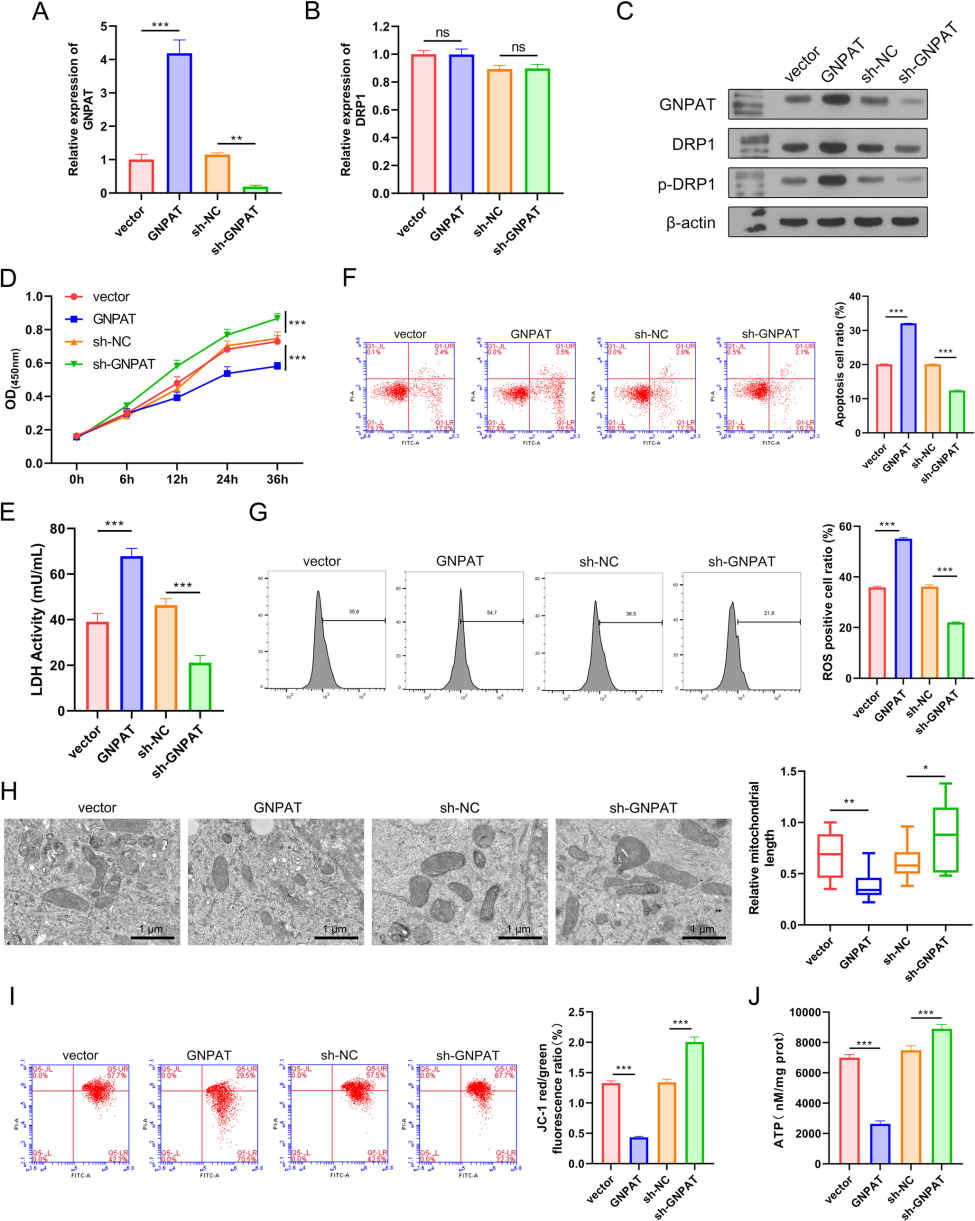
GNPAT increased DRP1 expression and promoted mitochondrial fission and dysfunction. The role of GNPAT in regulating mitochondrial fission and cell death was assessed by transfecting GNPAT overexpression and silencing plasmids into A549 cells stimulated with 5% CSE. (A, B) The levels of GNPAT and DRP1 mRNA expression in transfected cells. (C) A WB analysis determined the levels of GNPAT, DRP1, and p‐DRP1 protein expression in transfected cells. (D) The CCK‐8 assay was used to measure the cellular activity of A549 cells transfected with GNPAT overexpression and sh‐GNPAT plasmids. (E) LDH release was detected by the LDH activity assay. (F) Cell apoptosis in transfected cells was detected by flow cytometry. (G) The levels of total ROS were determined using a 2′, 7′‐dichlorofluorescein diacetate (DCFH‐DA) kit. (H) Mitochondrial morphology was examined by transmission electron microscopy. (I) Flow cytometry was used to assess mitochondrial membrane potential using JC‐1 dye. (J) ATP levels were measured using an ATP assay kit. *p < 0.05, **p < 0.01, ***p < 0.001. ns, no significant difference.

Subsequently, the role of GNPAT in regulating mitochondrial fission and cell death was assessed by transfecting GNPAT overexpression and silencing plasmids into A549 cells stimulated with 5% CSE. First, the levels of GNPAT and DRP1 mRNA expression were determined by qPCR. Results showed that GNPAT mRNA was y highly expressed in the GNPAT group and expressed at low levels in the sh‐GNPAT group when compared with the vector and sh‐NC groups (Figure 3A). Meanwhile, DRP1 mRNA expression was not influenced by either the GNPAT overexpression or silencing plasmids (Figure 3B). Next, the levels of GNPAT, DRP1, and p‐DRP1 protein were measured. GNPAT protein and GNPAT mRNA exhibited similar expression trends in the transfected cells (Figure 3C). Notably, the levels of DRP1 and p‐DRP1 protein expression showed a positive association with GNPAT, suggesting that GNPAT might regulate DRP1 at the post‐transcription level.

Cellular activity was inhibited by GNPAT overexpression and promoted by sh‐GNPAT when compared to effects produced by the vector or sh‐NC (Figure 3D). Cells exhibited higher LDH levels in the GNPAT group and lower levels in the sh‐GNPAT group, indicating enhanced cell damage caused by GNPAT (Figure 3E). Cell apoptosis was promoted by GNPAT overexpression and suppressed by sh‐GNPAT, as detected by flow cytometry (Figure 3F). The changes in ROS levels were consistent with cell apoptosis (Figure 3G). Mitochondrial length was significantly reduced by GNPAT overexpression and increased by GNPAT silencing (Figure 3H). When compared with the Vector and sh‐NC groups, the JC‐1 red/green fluorescence ratio was significantly reduced by GNPAT overexpression and increased by GNPAT silencing, revealing a reduction in mitochondrial membrane potential caused by GNPAT (Figure 3I). Additionally, overexpression of GNPAT significantly reduced the cellular ATP levels when compared with the vector (Figure 3J), while the ATP levels in the sh‐GNPAT group were significantly elevated when compared with those in the sh‐NC group.

### 3.4 GNPAT Enhanced DPR1 Protein Stability, Depending on USP30

**FIGURE 4:**
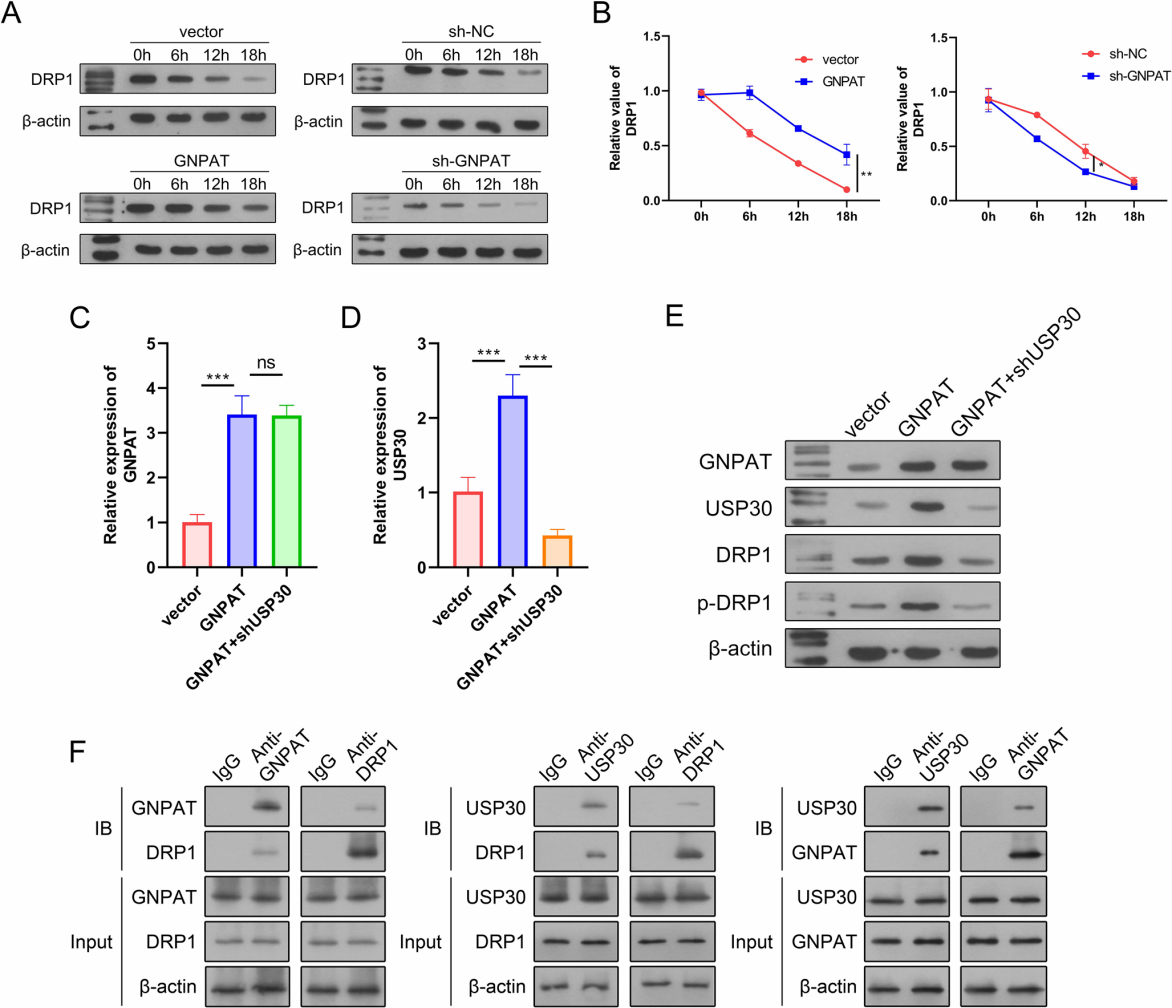
GNPAT enhanced DPR1 protein stability, depending on USP30. The interaction among GNPAT, USP30, and DRP1 was verified in vitro. (A, B) The levels of DRP1 expression in cells were measured after treatment with 50 mg/mL of CHX for 0, 6, 12, and 18 h, respectively. (C–E) The levels of GNPAT, USP30, DRP1, and p‐DRP1 expression were measured in the vector, GNPAT, and GNPAT + shUSP30 groups. (F) Co‐IP was performed to investigate the binding relationships among GNPAT, DRP1, and USP30. *p < 0.05. **p < 0.01. ***p < 0.001. ns, no significant difference.

Next, we conducted in vitro studies to further verify whether GNPAT promoted DRP1 protein expression by recruiting USP30. Transfected cells that overexpressed GNPAT and sh‐GNPAT were treated with 50 mg/mL of CHX for 0, 6, 12, and 18 h, and then co‐stimulated with 5% CSE. WB results showed that GNPAT overexpression notably enhanced the stability of DRP1 protein, while GNPAT knockdown reduced the stability of DRP1 protein (Figure 4A,B). To investigate the role of USP30 in regulating DRP1 expression, cells were transfected with an sh‐USP30 plasmid. No significant difference was observed between the GNPAT and GNPAT + shUSP30 groups regarding GNPAT expression (Figure 4C); however, USP30 expression was significantly lower in the GNPAT + shUSP30 group when compared with the GNPAT group (Figure 4D). A WB analysis showed that overexpression of GNPAT increased the levels of GNPAT, USP30, DRP1, and p‐DRP1 proteins, but with the exception of GNPAT, those levels were restored after simultaneous knockdown of USP30 (Figure 4E). A Co‐IP experiment showed that GNPAT, DRP1, and USP30 proteins could be enriched by the antibody of each other compared with IgG (Figure 4F). These results demonstrated that GNPAT and USP30 bound to DRP1, and GNPAT enhanced the stability of DPR1 protein, depending on USP30.

### 3.5 DRP1 Enhanced Mitochondrial Fission and Functional Damage, Which Were Reversed by GNPAT/USP30 Inhibition

**FIGURE 5:**
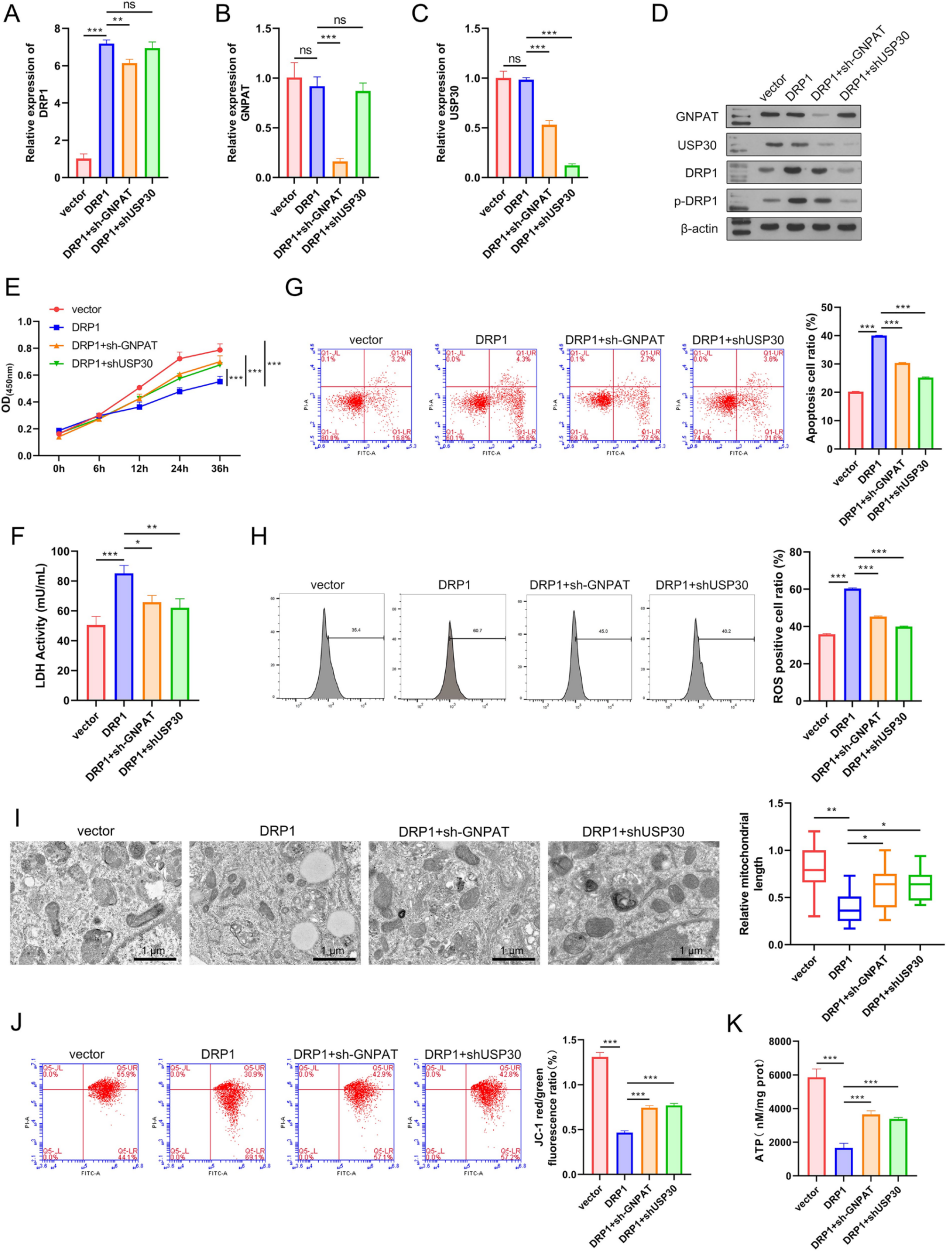
DRP1 enhanced mitochondrial fission and functional damage, which were reversed by GNPAT/USP30 inhibition. A549 cells were transfected with DRP1 overexpression, sh‐GNPAT, and shUSP30 plasmids, and gene function was tested in the CSE‐induced cell injury model. (A–C) The levels of DRP1, GNPAT, and USP30 mRNA in transfected cells were measured by qPCR. (D) The levels of DRP1, p‐DRP1, GNPAT, and USP30 protein expression in transfected cells were measured by Western blot. (E) Cellular activity in transfected cells was measured by the CCK‐8 assay. (F) LDH release was detected by the LDH activity assay. (G) Cell apoptosis in transfected cells was detected by flow cytometry. (H) The levels of total ROS were determined using a 2′, 7′‐dichlorofluorescein diacetate (DCFH‐DA) kit. (I) Mitochondrial morphology was examined by transmission electron microscopy. (J) Flow cytometry was used to assess mitochondrial membrane potential using JC‐1 dye. (K) ATP levels were measured using an ATP assay kit. *p < 0.05, **p < 0.01, ***p < 0.001. ns, no significant difference.

Subsequently, A549 cells were transfected with the DRP1, sh‐GNPAT, and shUSP30 plasmids, and their gene function was tested in the CSE‐induced cell injury model. First, the levels of GNPAT, DRP1, and USP30 expression were evaluated at both the mRNA and protein levels by qPCR and WB analyses. The qPCR analysis revealed that DRP1 was highly expressed in the DRP1 overexpression group when compared with the vector group (Figure 5A). GNPAT was expressed at significantly lower levels in the DRP1 + sh‐GNPAT group than in the DRP1 group (Figure 5B). When compared with USP30 expression in the vector group, its expression was significantly reduced in the DRP1 + GNPAT and DRP1 + shUSP30 groups (Figure 5C). Additionally, knockdown of GNPAT and USP30 expression had little effect on DRP1 mRNA levels. At the protein level, overexpression of DRP1 increased the levels of DRP1 and p‐DRP1 proteins, and both of those protein levels were reduced by knockdown of GNPAT or USP30 (Figure 5D). Overexpression of DRP1 significantly reduced cell viability and increased LDH levels when compared to transfection with the vector, but those effects were remarkably reversed by sh‐GNPAT or shUSP30 (Figure 5E,F). Furthermore, cell apoptosis and ROS levels were also significantly increased by DRP1 overexpression, and those effects were reversed by sh‐GNPAT and sh‐USP30 (Figure 5G,H). Mitochondrial length, mitochondrial membrane potential, and ATP levels were significantly reduced by DRP1 overexpression, but were restored by sh‐GNPAT and sh‐USP30 (Figure 5I–K). All the above results demonstrated that DRP1 enhanced mitochondrial fission and functional damage, which were reversed by GNPAT/USP30 inhibition.

## 4 Discussion

While our previous study revealed the role of GNPAT in COPD through regulating ferroptosis [8] its further mechanisms remained unclear. In this study, we explored whether GNPAT participated in COPD by regulating mitochondrial function via USP30/DRP1. Our experimental results showed that: (1) GNPAT and DRP1 proteins were highly expressed in COPD; (2) CSE and the mitochondrial fission inducer TA9 enhanced mitochondrial fission and dysfunction, and also promoted cell death; (3) GNPAT/USP30 increased the stability of the DRP1 protein, and thereby regulated mitochondrial fission and functional damage.

The ability of TA9 to induce mitochondrial fission has been proved in previous studies. Park et al. [23] demonstrated that TA9 promotes DRP1‐mediated mitochondrial fission leading to apoptotic cell death. A similar result was reported by Ahn et al. [24], who found that TA9 also facilitated blastocyst development by induction of DRP1‐dependent mitochondrial fission. In this study, we found that both CSE and TA9 increased the phosphorylation level of DRP1, enhanced mitochondrial fission and dysfunction, and also promoted cell apoptosis, suggesting the important role of mitochondria in COPD.

DRP1 is one essential protein that controls mitochondrial division [25, 26], by mediating the mitochondrial fission process [27]. It facilitates mitochondrial fission and modulates the response of lung epithelial cells to allergens [28]. Inhibition of DRP1 has been shown to block mitochondrial fission in fibroblasts via ROS/HIF‐1*α*, and thereby mitigate the progression of pulmonary fibrosis [29]. In COPD, Aravamudan et al. [30] demonstrated that CS triggers mitochondrial fragmentation and ROS production by increasing DRP1 expression. Tan et al. [31] revealed that CSE stimulated increased myostatin levels, elevated superoxide production, reduced mitochondrial membrane potential, significantly accelerated DRP1‐mediated mitochondrial fission, and facilitated apoptosis. Additionally, DRP1 overexpression was found to increase mitochondrial division, which was consistent with our present study. Zhao et al. [32] investigated the molecular mechanism of DRP1 in regulating COPD. Those investigators found that DRP1 was upregulated by Trophoblast cell surface antigen 2 (TROP2)/PTEN‐induced putative kinase 1 (PINK1) and mediated mitochondrial autophagy and apoptosis, thereby accelerating COPD progression in the elderly. In our present study, we also found that DRP1 was an essential molecule that enhanced mitochondrial fission and promoted cell apoptosis in COPD.

USP30 is a mitochondrial deubiquitinating enzyme located in the outer mitochondrial membrane (OMM) [33]. USP30 can be recruited to damaged mitochondria to rescue mitophagy deficiency. USP30 is crucial for various cellular processes, including apoptosis, pexophagy, mitophagy, and the pathways involved in lipogenesis and tumorigenesis [34, 35, 36]. Disruption of those pathways is linked to several physiological disorders, including pulmonary conditions, hepatocellular carcinoma, neurodegenerative diseases, and disorders related to peroxisome biogenesis [34]. USP30 has been shown to hinder mitochondrial quality control and worsen oxidative damage following traumatic brain injury [37]. Additionally, USP30 inhibitors have been explored as a treatment for pulmonary disorders. In this study, we found that GNPAT and USP30 bind to DRP1, enhancing the activity of DPR1 and thus regulating COPD progression in a USP30‐dependent manner.

The role of GNPAT has been rarely studied in COPD. Our previous study demonstrated that GNPAT mitigated CSE‐induced ferroptosis. In this study, we further investigated the molecular mechanism of GNPAT in COPD. We found that GNPAT increased DRP1 protein expression, the levels of cellular ROS, enhanced mitochondrial fission, damaged mitochondrial function, and promoted cell death. Here, we report those effects in COPD for the first time. The regulatory relationships among GNPAT, USP30, and DRP1 were previously reported in cancer. Gu et al. [20] demonstrated that GNPAT recruited USP30, which removed ubiquitin from and stabilized DRP1, thereby enhancing mitochondrial morphology and hepatocarcinogenesis regulation. A similar conclusion could be drawn from our experiments, as we revealed that GNPAT recruited USP30 and stabilized DRP1, thereby mediating mitochondrial morphology and cell apoptosis in COPD. These findings indicate that the interplay among DRP1, USP30, and GNPAT is critical for disrupting mitochondrial homeostasis and contributes to COPD progression.

Our previous research demonstrated the ferroptosis‐induced function of GNPAT [8], while this study identified a new effect of GNPAT on mitochondrial fission and cell apoptosis. In reality, there is substantial crosstalk between different forms of programmed cell death (PCD). These PCD mechanisms are intricately interconnected and influence and collaborate with one another instead of functioning in isolation [38]. Many genes have been verified to play a role in regulating both apoptosis and ferroptosis, such as *p53* and *Beclin‐1*. The ferroptosis inducer, erastin, was also found to induce apoptosis [39]. Furthermore, mitochondrial dynamics is also an important regulator of both ferroptosis and apoptosis [40]. Therefore, it is understandable that GNPAT regulates both ferroptosis and apoptosis due to its effects on mitochondrial dynamics.

## 5 Conclusion

Our present study investigated the molecular mechanism by which GNPAT/USP30/DRP1 regulates COPD by affecting mitochondrial morphology and cell apoptosis. We found that GNPAT recruits USP30 and stabilizes DRP1, thereby mediating mitochondrial fission and functional damage, which contribute to cell apoptosis in COPD.

## Ethics Statement

This research is approved by the Ethical committee of Hainan General Hospital (No. 2024‐236).

## Conflicts of Interest

The authors declare no conflicts of interest.

## Data Availability

All data are available from the corresponding author.
